# Chondroprotection of articular cartilage integrity: Utilizing ultrasonic scalpel and hyperosmolar irrigation solution during cutting

**DOI:** 10.1016/j.ocarto.2024.100499

**Published:** 2024-07-01

**Authors:** Nisreen Mohammed Al-Namnam, Aneta T. Luczak, Irene Yang, Xuan Li, Margaret Lucas, Andrew C. Hall, A. Hamish R.W. Simpson

**Affiliations:** a Department of Orthopaedics and Trauma, University of Edinburgh, Edinburgh, UK; b Royal Infirmary of Edinburgh, Edinburgh, UK; c James Watt School of Engineering, University of Glasgow, Glasgow, UK; d Deanery of Biomedical Sciences, University of Edinburgh, Edinburgh, UK

**Keywords:** Ultrasonic cutting, Articular cartilage, Chondroprotection, Osmolarity

## Abstract

**Objectives:**

Ultrasonic (US) cutting of cartilage in orthopaedic surgery has received little attention despite its potential to reduce chondrocyte death which could enhance cartilage repair. We aimed to investigate whether an ultrasonically-vibrating scalpel to cut human articular cartilage could reduce chondrocyte death, and to determine if hyper-osmolarity could provide chondroprotection during the procedure.

**Methods:**

A scalpel (no. 15) was mounted on an ultrasonic transducer to resonate at 35 ​kHz with 30 ​μm vibrational displacement. Thirty-six fresh human femoral cartilage samples were divided into four groups based on ultrasonic activation (US or non-US) and saline osmolarity (300 or 600 mOsm/L). Cell viability was assessed using a live/dead cell assay and analysed quantitatively by confocal microscopy. Histology illustrated tissue surface changes at the cut site.

**Results:**

The overall chondrocyte death percentage at both the US and non-US cut sites showed comparable results (p ​> ​0.05) in both osmolarities. However, the zone of chondrocyte death was reduced by 31 ​± ​5% and 36 ​± ​6%, respectively, when comparing US cutting at 300 mOsm/L and 600 mOsm/L to the control group (non-US cutting; 300 mOsm/L) (p ​< ​0.05). The width of the cut was consistent at both sites, regardless of the method of cutting.

**Conclusion:**

Cutting human cartilage with US in the presence of 300 or 600 mOsm/L media was chondroprotective compared to normal (non-US) scalpel cutting in 300 mOsm/L medium. These results suggest chondroprotection can be achieved while cutting using a US scalpel and raised osmolarity, potentially improving cartilage regeneration and repair following injury.

**What is already known on this topic**

Ultrasonic cutting devices are used for cutting bone in certain maxillofacial and spinal surgeries. They provide precise and controlled incisions. However, the impact of ultrasonic cutting on articular cartilage has not been studied yet.

**What this study adds**

Employing ultrasonic technology for cartilage cutting, along with hyperosmolar solution for irrigation during cutting, could potentially mitigate cartilage mechanical injury at the cut edge and facilitate lateral integration of graft and tissue engineered constructs, thereby presenting a promising novel avenue in the field of cartilage repair surgery.

**How this study might affect research, practice or policy**

There is a potential to enhance preventive measures for cartilage during cutting by using an ultrasonic scalpel with a higher osmolarity irrigation solution. This approach showed chondroprotection, with the potential to facilitate cartilage integrated repair.

## 1 Introduction

Hyaline cartilage has very limited repair and regeneration capacity mainly because it is an avascular tissue [1]. Chondrocytes represent approximately 1–5% of the volume in human hyaline cartilage and their viability is critical for the extracellular matrix to remain healthy and load-bearing, since they maintain a discrete maximum volume of surrounding matrix [2]. Chondrocyte death, as a result of mechanical injury such as cutting, probing, results in regions of extracellular matrix degradation at the wound edge that limits successful lateral integration is likely to be a starting point for further degeneration [3]. Moreover, prior research has shown that increasing the osmolarity of joint irrigation solutions could potentially act as a preventative measure against cartilage injury: increasing the osmolarity of 0.9% saline (285 mOsm) and Hartmann's solution (255 mOsm) to 600 mOsm resulted in a decrease in situ chondrocyte death in a bovine model of mechanical cartilage injury [4]. Another recent experimental study demonstrated that it was possible to reduce the zone of cell death at the cartilage edge created during cutting by using high (600 mOsm/L) rather than normal osmolar (300 mOsm/L) solutions [5,6]. It showed that exposing articular cartilage to a hyperosmolar solution (600 mOsm/L) reduced chondrocyte death associated with scalpel-induced injury in both in vitro and in vivo cartilage injury models [5]. The zone of chondrocyte death that occurs due to cartilage cutting is likely to restrict or even prevent successful lateral integration across the interface between host and repair tissues [7]. Thus, a reduction of injury associated with cutting is desirable, to reduce the zone of chondrocyte death, promote integrative cartilage repair, and thereby have the potential to improve patient outcomes [8].

Functional limitations and the potential for joint degeneration are common consequences of articular cartilage defects. Therefore, it is crucial to address these defects during joint preservation surgery. Traditionally, cartilage defect preparation involves the use of a sharp scalpel. Nevertheless, achieving successful reconstruction of these defects remains challenging, particularly in terms of obtaining mechanically resilient hyaline cartilage-like tissue formation at the injury site [[7], [8], [9], [10]]^.^

Ultrasonic cutting devices are used for cutting bone, for example in maxillofacial and spinal surgeries, due to their low cutting force and high precision [11]. These characteristics could potentially reduce damage to the chondrocytes and matrix when cartilage is cut which ultimately may improve integrative repair between new cartilage and the surrounding host articular tissue. Thus, the aim of this study was to investigate whether the zone of cell death at the wound edge of injured human articular cartilage could be decreased by using an ultrasonic scalpel, and if this reduction could be further enhanced by employing a hyperosmolar irrigating solution.

## 2 Materials and methods

### 2.1 Design of the ultrasonic scalpel

**Fig. 1:**
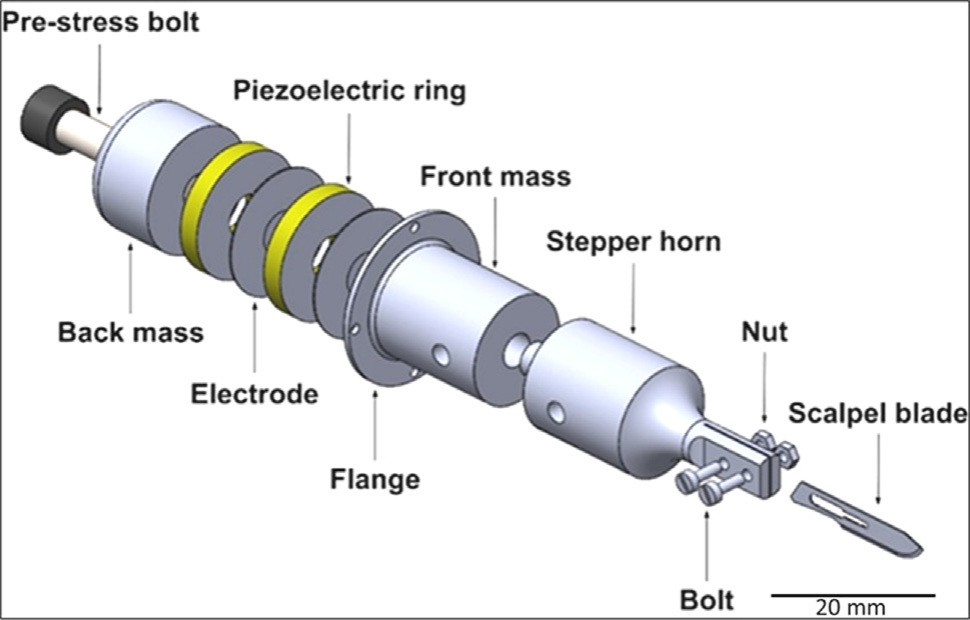
Expanded view of the ultrasonic (US) scalpel structure of the ultrasonic scalpel based on a Bolted Langevin Transducer.

The design of the ultrasonic scalpel was based on the configuration of a bolt Langevin transducer (Fig. 1).

The ultrasonic scalpel was modelled and tuned using finite-element analysis software package (ABAQUS) to the second longitudinal mode (L2) at around 35 ​kHz. A half wavelength transducer consisted of a pair of piezoelectric rings (PIC181, propidium iodide (PI) Ceramic; Germany, outer diameter: 30 ​mm; inner diameter: 10 ​mm; thickness: 5 ​mm, material properties are shown in Table 1 in the supplementary file) and sandwiched between a cylindrical back mass and a cylindrical front mass, were fabricated by applying pre-stress to the bolt. A step shape horn with a slot, produced to grip the scalpel blade by means of two sets of bolts and nuts, was tuned to the half wavelength, and attached to the half wavelength ultrasonic transducer via a threaded stud to form a full wavelength system. A flange was produced at the rear side of the front mass, where the longitudinal displacement nodal point is located, to hold the ultrasonic scalpel system in a casing with minimal energy loss. Titanium grade 5 alloy (Ti–6Al4V) was used for the back mass, front mass, and the step shape horn. The pre-stress bolt was constructed from standard A4 tool steel, the electrodes were made of copper, and the scalpel blade was crafted from surgical grade carbon steel. Material properties of the components of the ultrasonic scalpel are presented in Table II in the supplementary file. Characterization and experimental modal analysis of the ultrasonic scalpel can be found in the supplementary.

### 2.2 Cartilage explant preparation and testing

#### 2.2.1 Study design

The study was approved by the ethical committee of the institutional review board (ethics approval number: IRB00001462) and prior informed consent was obtained from all patients.

Three femoral heads were obtained from hip replacement surgeries of three patients (one femoral head from each patient), diagnosed with osteoarthritis grade 1, at the Royal Infirmary of Edinburgh, UK. The patients ranged from 69 to 80 years old, with an average weight of 67 ​kgs, 2 female and one male.

The cartilage samples were postoperatively placed into a sterile container containing serum-free Dulbecco's modified Eagle's medium (320 mOsm/L) (DMEM 41965–039; Invitrogen, Paisley, UK) and available immediately for experiments.

#### 2.2.2 Cartilage preparation

The samples were collected from the areas of the femoral head cartilage with less cartilage degradation exhibited osteoarthritis grades 1 according to OARSI Osteoarthritis Cartilage Histopathology Assessment System [12]. A total of 36 samples, with 12 samples obtained from each of the 3 patients involved in the study were collected. These 36 samples were then randomly allocated into four groups, each containing 9 samples based on the osmolarity of the solution used and the type of cutting applied (ultrasonic, US, or non-ultrasonic, non-US): US cutting in a hyperosmolar saline (US-600 mOsm/L), US cutting in normal osmolarity saline (US-300 mOsm/L), non-US scalpel (non-US) cutting in a hyperosmolar saline (non-US 600 mOsm/L) and non-US cutting in normal saline (non-US 600 mOsm/L).

#### 2.2.3 Cartilage mechanical injury

**Fig. 2:**
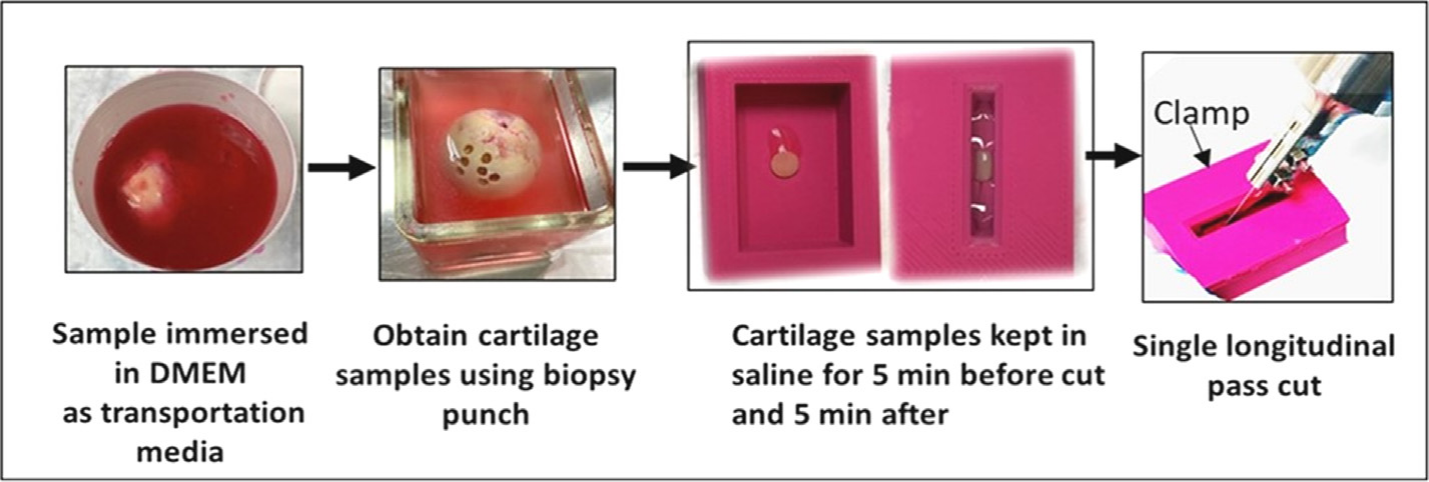
Human tissue sample collection and cutting technique.

The articular cartilage sample was held in a custom 3D printed clamp (Fig. 2) to stabilise the cartilage during cutting. Saline osmolarity (275 mOsm/L) adjusted with sucrose (97 ​g; Sigma-Aldrich, Poole, UK, per 1 ​L of normal saline) and measured using an osmometer (Fiske Micro-Osmometer 210). Cartilage explants were incubated in saline solutions with different osmolarities: a 0.9% normal saline solution with 600 mOsm/L (from the addition of sucrose) as a hyperosmolar solution and 300 mOsm/L as a normal osmolar solution, both before and after cutting, 5 ​min each at 25 ​± ​2 ​°C. Each sample was cut using a single longitudinal pass of a fresh (after each three performed cuts) no. 15 scalpel blade mounted on the ultrasonic transducer (ultrasonic unit powered off (Non-US Scalpel) or powered on (US scalpel) (Fig. 1) driven using an ultrasonic generator (PDUS210 Piezodrive).

### 2.3 Investigation

#### 2.3.1 Live and dead assay

In situ chondrocyte viability was determined by incubating the human cartilage explants with 5-chloromethyl-fluorescein diacetate (CMFDA) (Abcam ab145459) and PI (Invitrogen P3566) for 45 ​min, both at 10 ​μM in DMEM, to label live and dead cells, respectively. The explants were then fixed in 4% formalin for 1.5 ​h at 21 ​°C (Fisher, Leicester, UK), washed twice in phosphate-buffered saline (Invitrogen, Paisley, UK), and stored in phosphate-buffered saline at 4 ​°C.

#### 2.3.2 Imaging

Using a confocal laser scanning microscope (Leica TCS SP8, Germany), consecutive series of optical sections in the axial plane from mechanically-injured cartilage were acquired at 10 ​μm intervals between each image in the stack. A standard multitrack procedure, which involves sequentially scanning different fluorescent channels to detect multiple fluorophores within the same sample, was used for fluorescence detection within individual optical sections. This included the use of argon (Ex ​= ​488 ​nm) and helium-neon (Ex ​= ​543 ​nm) lasers. This process incorporated band-pass filters ranging from 500 to 550 ​nm and long-pass filters, >560 ​nm, to detect the two fluorophores emitted by CMFDA and PI respectively, facilitating the visualization of labelled live and dead cells. Laser power, detector gain, and sensitivity were manually adjusted before acquiring each image to capture the optimal signal from each section without pixel saturation. A 3-dimensional reconstruction of all optical sections was then created using Imaris™ (Oxford Instruments). The region of interest (ROI) applied was 200 ​μm from the centre of the scalpel injury (x-axis) ​× ​819 ​μm (y-axis) ​× ​200 ​μm (z-axis). The chondrocyte death after injury was then determined within the ROI. Live and dead cells were identified in the green and red channels respectively, by thresholding volumetric pixel intensity. The percentage cell death was calculated in the ROI. The width of the injury was measured at 100 ​μm intervals along the y-axis in the confocal laser scanning microscope images. In all samples, 8 widths per projection were taken to calculate the mean width.

#### 2.3.3 Histology

Cartilage explants were fixed in 10% formalin, immersed in 30% sucrose solution (w/v) for cryoprotection, and embedded and frozen into Optimal Cutting Temperature embedding Matrix. Samples were cut into 7 ​μm coronal sections in a cryostat (Bright Instruments- Oxford Instruments). Sections were then stained with Harris Hematoxylin (7 ​min), washed in distilled water, and incubated in Scott's Tap Water Substitute for 15 ​min (Sodium Bicarbonate, 20 g/L (Sigma S6297) and Magnesium Sulfate • 7H2O, 200 g/L (Sigma M7506) as standard operating procedure (Sigma Aldrich). Following this, sections were placed in Eosin solution (5 ​min), washed briefly in deionised water, and immersed in potassium alum (Fisher 10647411) (0.5%; 5 ​min). Sections were finally washed in tap water before mounting and being examined microscopically (Leica brightfield microscope, Japan).

### 2.4 Statistics

All statistical analyses used a 95% confidence level and were conducted using IBM SPSS Statistics for Windows (Version 24.0., IBM Corp., Armonk, NY, USA). The ANOVA test was used to reveal if there was a significant difference between the four groups in terms of cell death and the zone of death. Tukey's post-hoc tests were used to identify the specific group differences.

## 3 Results

### 3.1 Chondrocyte viability and the zone of death

**Fig. 3:**
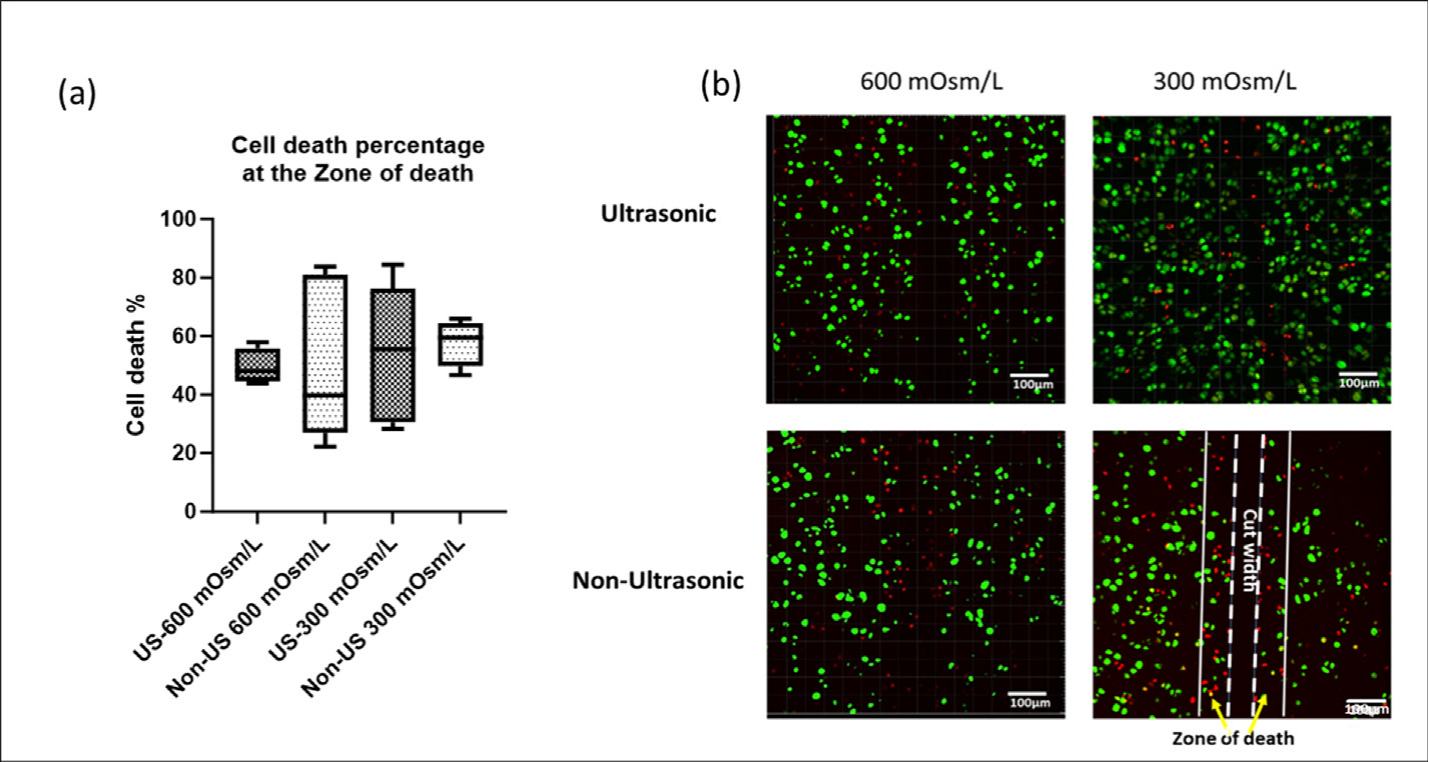
Marginal cell death as a function of medium osmolarity and method of the cutting. (a) Percentage cell death at the cut site as a function of medium osmolarity using US scalpel and non-US cutting, data represent the mean ​± ​SD from three replicates (b) Axial view of in situ chondrocyte death associated with human femoral cartilage cut, dead cells (red) and live cells (green). The area between the white dashed line and white arrows indicated the zone of cell death extending from the incision's surface (dashed lines) toward the tissue (white lines). The space between the two white dashed lines represents the cut width. (For interpretation of the references to color in this figure legend, the reader is referred to the Web version of this article).

The effect of ultrasonic cutting on cartilage was examined by comparing the percentage of chondrocyte death resulting from cartilage cuts made with the ultrasonic scalpel powered on (US-scalpel) to those made with the scalpel powered off (non-US scalpel). The percentage cell death at the margin of the cut site (zone of death) after using the US scalpel was comparable to that observed with the non-US scalpel (p ​> ​0.05) (Fig. 3 (a)) for both osmolarities. A higher cell death was noted at 300 mOsm/L compared to 600 mOsm/L. Fig. 3 (b) displays the dead cells confined at the margin of the cutting edge within 0–45 ​μm on the US-scalpel cut site and within 0–76 ​μm on the non-US cut site, extending from the cut margin into the tissue depth. These findings suggest that the US scalpel causes a similar level of cell death at the cut margin compared to the non-US, but the cell death is more localized with the US scalpel.

**Fig. 4:**
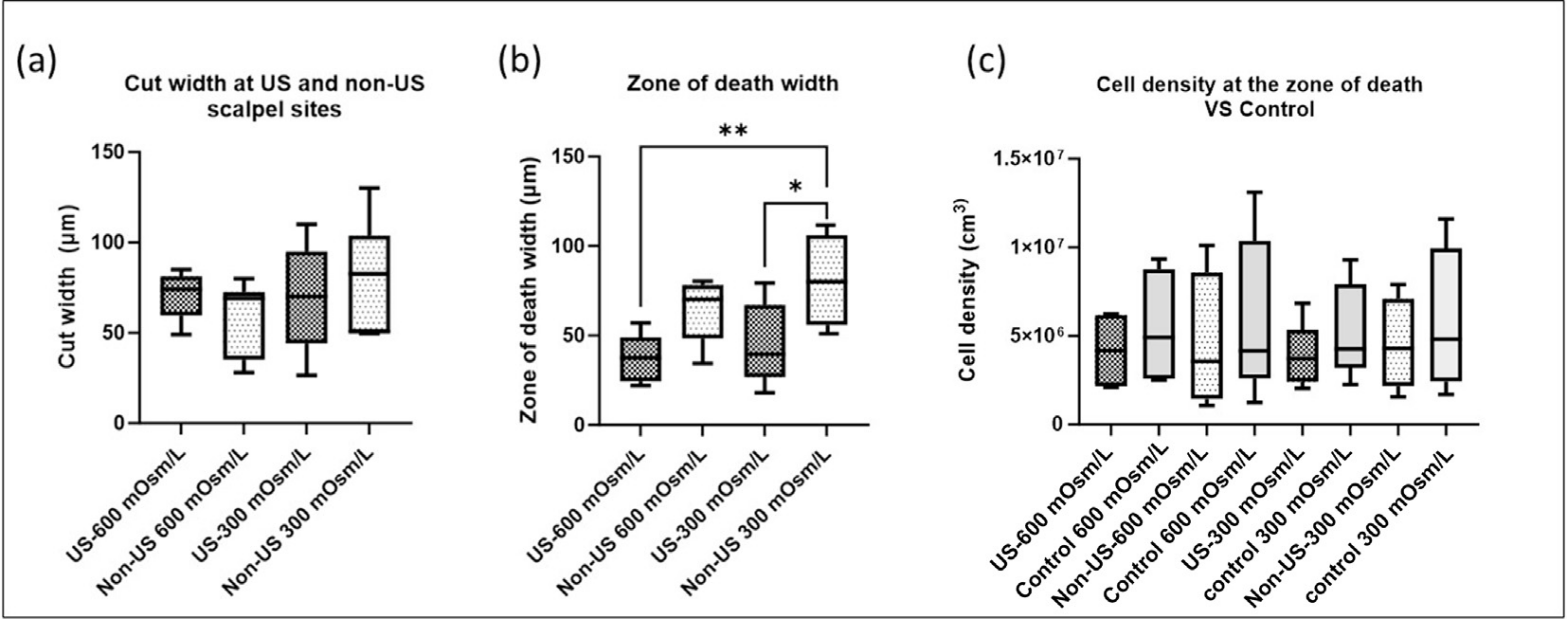
The difference in the cut width, width of the zone and chondrocytes density at both US and non-US cut sites in both medium osmolarities. (a) Cut width made by a No. 15 scalpel, whether using ultrasonic- (US) or non-US scalpel (b) Width of the zone of chondrocyte death at both US and non-US cut sites in both medium osmolarities (c) The difference in cell density between the zone of death and intact cartilage for the same volume, data represent the mean ​± ​SD.

There was no significant difference in the cut width between the control and powered on US scalpel groups, whether at 300 or 600 mOsm (Fig. 4 (a)). However, A significant difference (p ​< ​0.05) in the width of the zone of cell death was observed, with the non-US cut site at 300 mOsm/L showing a wider zone compared to both the US scalpel cut site at 600 mOsm/L and 300 mOsm/L, indicating an increase of 90% and 69% respectively (Fig. 4 (b)). The average width of the zone of cell death was significantly narrower (p ​< ​0.05) by 31 ​± ​5% at 300 mOsm/L when using the US scalpel compared to the non-US, and by 36 ​± ​6% for the US scalpel at 600 mOsm/L compared to the non-US at 300 mOsm/L. This demonstrated that the US scalpel group, in combination with the hyperosmolar solution, resulted in the smallest zone of chondrocyte death compared to the other groups.

To investigate the effect of cutting using US and non-US on cell injury, cell density was measured within the ROI (819 ​× ​400 ​× ​200 ​μm), the zone of death, and outside the zone of death (excluding the cut area in all measurements), compared to intact cartilage. The results, shown in Fig. 4 (C), indicated no significant difference. This observation suggests that the identified injury might not have resulted in the displacement of cells during the cutting process.

### 3.2 Histology

**Fig. 5:**
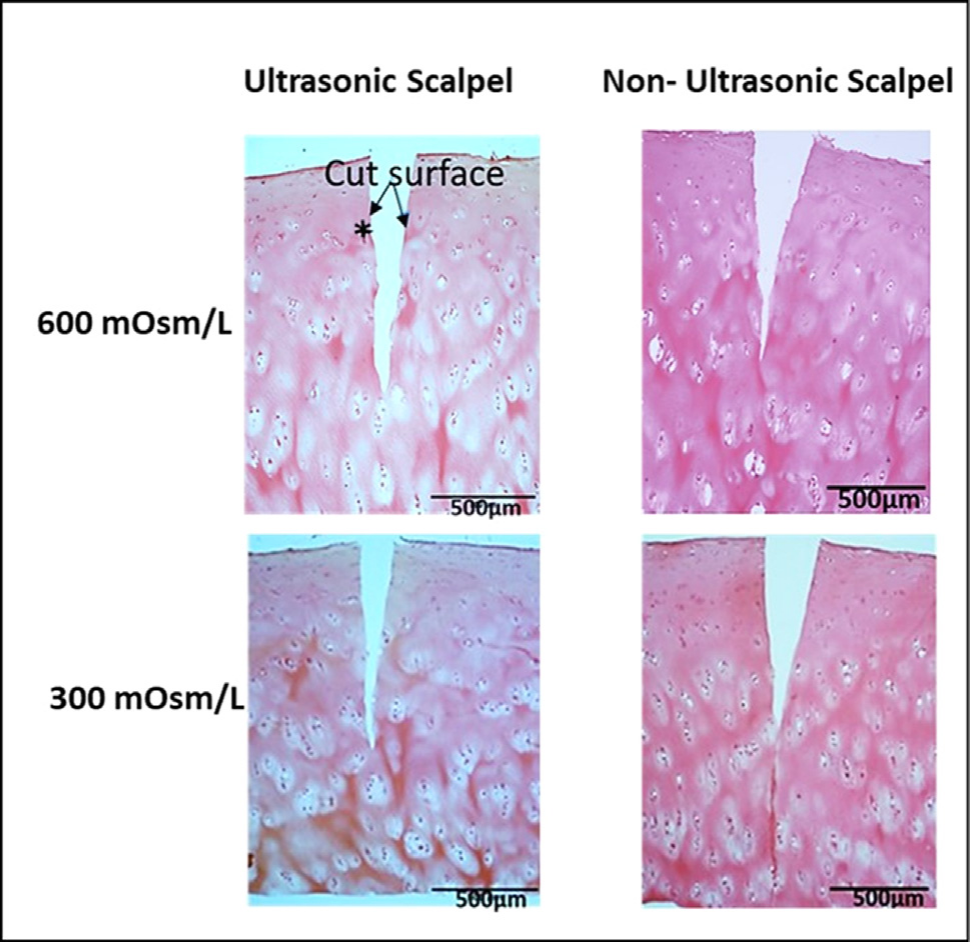
Histological section of articular cartilage explant, stained with hematoxylin and eosin, shows the microscopical features of the cartilage at the cut sites. Smooth cut surfaces and many chondrocytes exhibited with clearly delimited nuclei were observed (∗) in both US and non-US cut sites cells.

On histological examination, the cut surfaces of the cartilage were visually noted to be smooth, indicating that the US scalpel did not cause noticeable cartilage destruction (Fig. 5). Additionally, the similarity observed in both the depth and width of cuts suggests that the mechanical injury produced is comparable when using both US and non-USs for cartilage cutting. Abundant chondrocytes with clearly delimited nuclei were observed along the depth (1 ​± ​0.2 ​mm) of the US and non-US cut.

## 4 Discussion

US and non-US-cut explants from human femoral head cartilage were used to study the effect of ultrasonic cutting and medium osmolarity on chondrocyte viability to determine if the width of zone of death at the cut site could be significantly reduced. Our findings demonstrated that cartilage cut in a hyper-osmolar solution with an US scalpel had a narrower zone of chondrocyte death than cartilage samples cut in a normal osmolar solution with a non-US scalpel (Fig. 4 (b)). Moreover, when comparing the US and non-US groups used within the same osmolar solution, whether at 600 or 300 mOsm/L, the US groups consistently exhibited comparable cell death with a narrower zone of death. This indicates that the US scalpel provides an additional benefit for cartilage protection which could potentially enhance lateral integration of new and existing cartilage during reconstructive procedures.

The duration that the explants were exposed to a medium with altered osmolarity (∼5 ​min) was determined from previous studies that established that chondrocyte volume changes during this period [5]. In the previous study, it was observed that a short-term exposure of articular cartilage to a hyperosmolar solution (600 mOsm/L), led to a higher long-term viability of chondrocytes when compared to solutions with lower or normal osmolarity [5]. In our study, the duration used for exposing the explants to the medium was determined based on previous studies that established chondrocyte volume changes during this period.

The composition and organisation of the extracellular matrix in articular cartilage is critical to its biomechanical functionality [[13], [14], [15]]. The primary functions of the cartilage matrix include withstanding mechanical stress through the organization of collagen fibrils. Aggrecan, a constituent of the cartilage matrix, plays a role in its resistance to compression [13]. Studies by Liu et al. have demonstrated that mechanical stress can influence the phenotype of chondrocytes, leading to a downregulation of collagen II (COL II) expression and an upregulation of Matrix metallopeptidase 13 (MMP13) expression [14]. Certain studies investigate the relationship between proteoglycan loss, collagen damage due to overloading, and cyclic loading in articular cartilage. These studies examine how the degree of matrix damage and chondrocyte death in mechanically stressed cartilage explants varies depending on the loading rate [15,16]. By employing ultrasonic tool vibration as a cutting technique for cartilage, there is a potential to reduce the impact of mechanical stress on cartilage cells due to the low applied force needed during cutting.

Previous work has established that in situ chondrocytes are highly sensitive to their osmotic environment [10]. Reducing osmolarity below that experienced in the matrix will cause cell swelling whereas raising osmolarity will cause cell shrinkage [7]. As a consequence of their greater susceptibility to osmotic responses, chondrocytes that swell due to hypo-osmolar solutions are more prone to mechanical injury [17]. Furthermore, changes to chondrocyte volume can initiate intracellular signalling cascades which potentially interact with other pathways such as those that generate reactive oxygen species, which leads to mitochondrial oxidative damage and cell death [18]. Our findings show that the extent of chondrocyte death at the wound site decreased in both US and non-US cuts with increasing osmolarity (Fig. 3 (a)). The observed significant reduction in chondrocyte death due to increased osmolarity is consistent with findings from a previous study [5]. This study reported a decrease in chondrocyte death in rat articular cartilage following scalpel-induced injury as the medium's osmolarity was raised, observed in both in vitro and in vivo models of cartilage injury [5]. However, our results did not demonstrate a significant reduction in cell death, possibly because the samples were collected from osteoarthritic patients with varying levels of both cell death and cartilage integrity.

To determine the actual impact of using the US scalpel on cell death, the cell density in both the zone of death and intact cartilage was measured, showing no significant difference (Fig. 4 (c)). We suggest that the injury didn't cause extensive cell displacement. Consequently, the tissue appears to have maintained a consistent cellular distribution despite the cartilage split created by the cut.

In the context of cartilage injury, degradation of proteoglycans may prompt the migration of chondrocytes from undamaged areas to distant injury sites [19]. Clinical attempts at grafting osteochondral cylinders to fill cartilage defects sometimes fail to lateral integration of the graft cartilage with the adjacent host cartilage, creating a risk of further progressive cartilage degeneration over time [20]. The presence of a death zone resulting from mechanical interventions for example, cutting, significantly influences the healing process, as nearby cells proliferate and migrate to facilitate tissue regeneration [7,8,21]. However, the death zone disrupts the extracellular matrix, necessitating remodelling to restore structural integrity. Depending on the severity of damage, this can lead to increased susceptibility to degenerative changes and affect tissue integration. Understanding these healing mechanisms is crucial for developing effective strategies to enhance tissue repair and regeneration.

### 4.1 Clinical implications

To achieving successful cartilage reconstruction and improve patient outcomes, it is desirable to reduce injury associated with articular cartilage cutting, minimize the zone of chondrocyte death, and promote integrative cartilage repair. This study demonstrated the potential of using an ultrasonically vibrating scalpel and increasing the osmolarity of irrigation solutions to reduce the zone of cell death at the cartilage edge created during cutting, suggesting chondroprotective effects.

### 4.2 Limitation

It is important to note that although this study provided proof of principle that an US scalpel reduced the zone of death and increasing the osmolarity reduced chondrocyte death, it still has certain limitations. The cartilage tissue for the study, was collected from the macroscopically grade 1 regions of the osteoarthritic articular surface of the femoral heads. The exact force exerted during cutting was not measured, potentially leading to variations of force applied during cutting due to the lack of monitoring. Nevertheless, this approach attempted to emulate the conditions encountered clinically. Additionally, the force used with the US scalpel was minimal since the ultrasonic cutting blade utilizes ultrasonic vibration to reduce the cutting force required during the cutting process.

Our study demonstrated proof of principle that the use of the US-scalpel with increase osmolarity reduced the zone of death and chondrocyte death. Specifically, our study only assessed chondrocyte viability as a single outcome measure. Future research should include assessing the influence of US-scalpel on full-thickness cartilage (osteochondral) ex vivo and in vivo using animal models to evaluate other outcomes such as gene expression, the healing process, and the effects of US vibration on different layers of the cartilage.

## 5 Conclusion

Cutting cartilage with an ultrasonic scalpel resulted in a narrower zone of death compared to non-ultrasonic scalpel in both osmolarities. Applying US vibration to a standard surgical blade and increasing the osmolarity of irrigation solutions in cartilage cutting was chondroprotective, which potentially might improve integrative repair between new cartilage and the host articular cartilage.

## Author contributions statement

All authors have made substantial contributions to either; research design, acquisition, analysis and interpretation of data; or drafting of the paper and revising it critically.

All authors have read and approved the final submitted manuscript.

## Declaration of competing interest

The authors declare that they have no known competing financial interests or personal relationships that could have appeared to influence the work reported in this paper.
