# Supplementary material for: Chondroprotection of articular cartilage integrity: Utilizing ultrasonic scalpel and hyperosmolar irrigation solution during cutting
Source: Osteoarthr Cartil Open. 2024 Jul 1;6(3):100499. doi: 10.1016/j.ocarto.2024.100499 (PMC11284703; doi:10.1016/j.ocarto.2024.100499)
Supplement: Multimedia component 1 [file mmc1.docx]

**ULTRASONIC SCALPEL**

**Design of the ultrasonic scalpel**

**Table 1** Material properties for the PCI181 Piezoelectric ring

| Density *ρ* [kg/m^3^] | 7850 |
| --- | --- |
| Relative permittivity $\varepsilon_{11}^{T}$ | 1224 |
| Relative permittivity $\varepsilon_{33}^{T}$ | 1135 |
| Piezoelectric charge coefficient *d*_31_ [pC/N] | -108 × 10^-12^ |
| Piezoelectric charge coefficient *d*_33_ [pC/N] | 253 × 10^-12^ |
| Piezoelectric charge coefficient *d*_15_ [pC/N] | 389 × 10^-12^ |
| Elastic compliance coefficient *S*_11_*^E^* [m^2^/N] | 1.175 × 10^-11^ |
| Elastic compliance coefficient *S*_33_*^E^* [m^2^/N] | 1.411 × 10^-11^ |
| Poisson’s ratio *ν* | 0.35 |
| Coupling coefficient *k*_33_ | 0.66 |
| Mechanical quality factor *Q* | 2200 |

**Table 2** Material properties of the components of the ultrasonic scalpel

|  | **Ti6Al4V** | **Copper** | **A4 tool steel** | | **Carbon steel** |
| --- | --- | --- | --- | --- | --- |
| Density ρ [kg/m3] | 4430 | 8900 | 8000 | 7870 | |
| Young’s modulus E [GPa] | 109 | 110 | 210 | 200 | |
| Poisson’s ratio ν | 0.313 | 0.37 | 0.29 | 0.29 | |
| Acoustic impedance [Pa.s/m×106] | 27.69 | 42.02 | 44.66 | 46.47 | |

**Characterization**

The fabricated ultrasonic scalpel was characterised using electrical impedance analysis (IA) and experimental modal analysis (EMA). The displacement amplitude at the tip of the blade was measured using a commercial ultrasonic resonance tracking unit (PDUS210 Piezodrive).

1. **Electrical impedance analysis**

 Electrical impedance measurements were performed using an impedance analyser (Agilent 4294A). A swept signal of 1 V peak-to-peak over a bandwidth covering the frequency of interest was applied (in this case, frequency range was centred on 35 kHz), and the impedance spectrum was measured. The effective electromechanical coupling coefficient, *K_eff_*, was calculated from the impedance spectrum data using the following equation [2]. This parameter provides a measure of the ultrasonic scalpel conversion efficiency from electrical energy to mechanical vibrations:

$k_{eff}^{2}=\frac{f_{a}^{2}-f_{r}^{2}}{f_{a}^{2}}$ (1)

Where *f_a_* is the antiresonance frequency and *f_r_* is the resonance frequency. Mechanical Q factor can also be calculated from the impedance spectrum, which is an indicative factor of the ultrasonic scalpel’s potential to achieve high displacement amplitudes and low losses.

1. **Experimental modal analysis (EMA)**

EMA is performed by measuring the frequency response functions (FRFs) from a grid of vibration response measurement points on the surface of the ultrasonic scalpel, from which the modal parameters (frequency, damping, and mode shape) were extracted [1]. A white noise excitation signal of 15 V_rms_ was generated by a signal generator (Quattro, Data Physics) and amplified by a power amplifier (QSC RMX 4050HD), before being supplied to the ultrasonic scalpel. A 3-D laser Doppler vibrometer (CLV3000, Polytec) was used to measure three orthogonal components of the vibrational velocities from the grid points. Data acquisition and processing software (SignalCalc, Data Physics) was used to calculate the FRFs from the excitation and response signals and then to apply curve-fitting routines to extract the magnitude and phase data. Finally, the measured FRFs were exported to modal analysis software (ME’scopeVES, Vibrant Technology) to extract modal parameters.

To understand the vibrational behaviour of the scalpel blade, which could be critical to the cartilage cutting process, a scanning 3-D laser Doppler vibrometer (MSA-100, Polytec) was used, and the scanning area was focused around the profile of the scalpel blade.

1. **Displacement amplitude measurement**

Displacement amplitude at the blade of the ultrasonic scalpel excited in resonance at different excitation levels was measured using a 1-D laser Doppler vibrometer (OFV 303, Polytec), which is excited by a commercial ultrasonic resonance tracking unit (PDUS210 Piezodrive). In this study, the current control strategy was employed to track the series resonance of the ultrasonic scalpel, approximately proportional to the displacement amplitude of the blade. Different reference current levels are prescribed in the Piezodrive software, and the displacement amplitudes of the blade were subsequently measured.

**RESULTS AND DISCUSSION**

**Ultrasonic scalpel characterization**

To understand the electromechanical responses of the fabricated US transducer, as a validation of the theoretical design, the initial studies were performed to characterise the US transducer when attached to a standard scalpel blade.

**Electrical impedance**

The measured impedance of the half wavelength ultrasonic transducer at various levels of applied pre-stress to the bolt illustrated the required value of the pre-stress to achieve the electrical stability of the transducer (Fig. 1 (a)). The change in the resonance and anti-resonance frequencies reduced significantly as the torque was increased. The electrical stability was reached at an ultimate 30 Nm (Newton-metre) torque, with a resonance frequency close to the predicted 35 kHz value from the FEA model (Fig. 1 (b)).


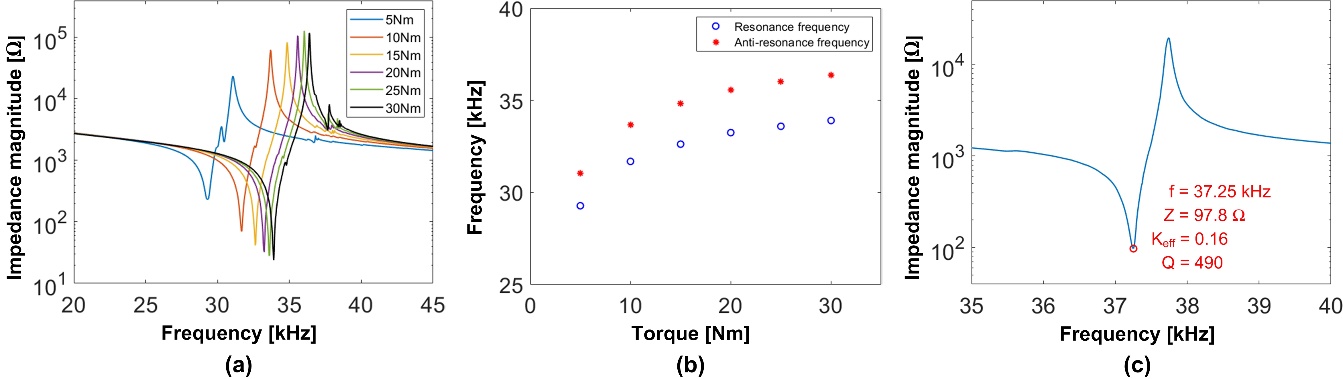


Fig 1. Electrical impedance characteristics of the ultrasonic scalpel: (a) impedance of the half wavelength ultrasonic transducer with different pre-stress, (b) resonance and anti-resonance frequencies as a function of torque, (c) impedance of the full wavelength ultrasonic scalpel

The electrical stability of the half wavelength ultrasonic transducer was achieved at a 30 Nm torque with a resonance frequency close to 35 kHz, the desired value. However, the full wavelength US scalpel presents a roughly 37 kHz resonance frequency, demonstrating the effect of the scalpel and mechanical fasteners added to the horn (Fig. 2). However, the predicted vibration modeshape of the US scalpel shows a good consistency with the experimental measurement. Due to the asymmetry of the blade, the detailed characterisation of the motion shows a combined longitudinal and lateral movement. A potentially greater than 30 µm peak-to-peak amplitude at the blade can be achieved.

**Experimental modal analysis**

The vibration modes and resonance frequencies predicted in FEA and extracted from EMA presented in Fig. 2 (a) and (b) showed a close agreement in the nodal plane locations and gain values (both around 20), indicating the generated ultrasonic vibration from the piezoelectric elements will be amplified by this number^1^. In respect of the vibration modeshape of the scalpel blade, the vibration presented a combined motion in the Y (lateral) and Z (longitudinal) directions (Fig. 2 (c)).


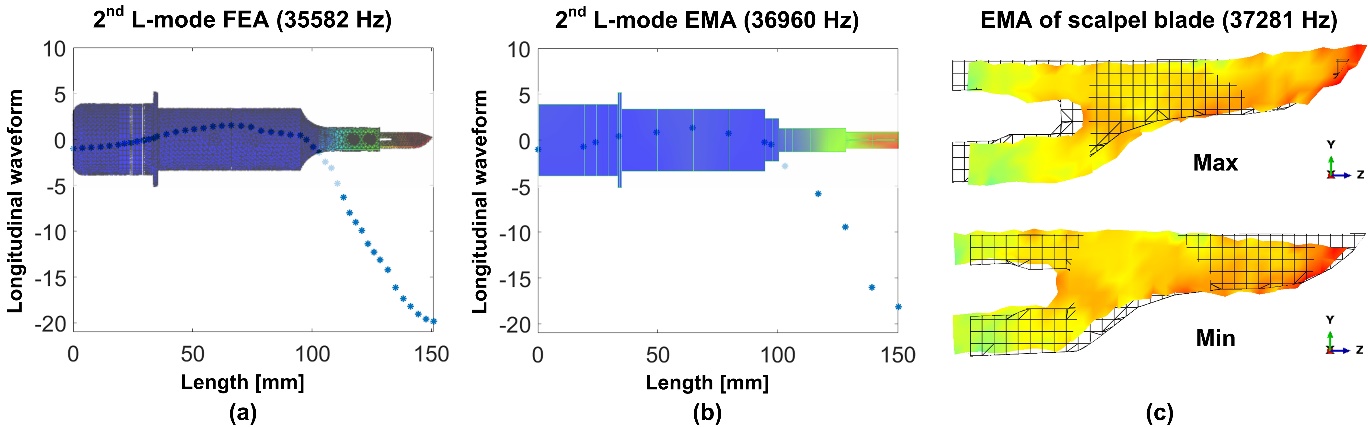


Fig 2. finite-element analysis (FEA) predicted and experimental modal analysis (EMA) measured normalized L2 mode waveform of the ultrasonic scalpel: (a) FEA prediction, (b) EMA measurement, (c) modeshape of the blade

**Displacement amplitude**

Displacement amplitude of at the tip of the blade of the ultrasonic scalpel was measured with varying prescribed levels of current of the Piezodrive. Amplitude increased linearly from slightly under 5 µm to 30 µm peak-to-peak with a current increasing from 0.05 A to 0.4 A (Fig. 3). The amplitude could potentially continue to increase, however, the 1-D LDV measurement has experienced difficulty of focusing at 0.4 A current, due to the thin and sharp nature of the blade, causing the laser reflective tape to fall off the blade.


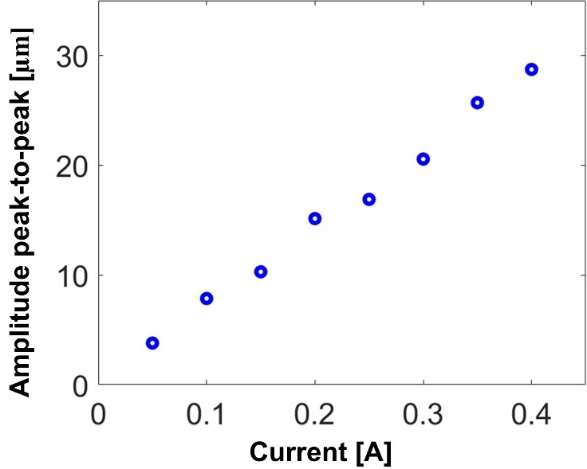


**Fig 3.** Amplitude-current characteristic of the ultrasonic scalpel

**Ref.**

1. Avitabile P. Experimental modal analysis. Sound and vibration. 2001;35(1):20-31.

2. Caronti A, Carotenuto R, Pappalardo M. Electromechanical coupling factor of capacitive micromachined ultrasonic transducers. The Journal of the Acoustical Society of America. 2003;113(1):279-88.
